# The association between digestion-resistant and bioactive peptide content of dairy products and bladder cancer: a case-control study

**DOI:** 10.1186/s41043-025-01071-2

**Published:** 2025-09-30

**Authors:** Atiyeh Sadat Hosseini, Seyyed Mostafa Jalali, Zainab Shateri, Marzieh Shoja, Milad Rajabzadeh-Dehkordi, Maede Makhtoomi, Melika Hajjar, Bahram Rashidkhani, Mehran Nouri

**Affiliations:** 1 Faculty of Nutrition and Food Technology, Shahid Beheshti University of Medical Sciences, Tehran, Iran; 2 Department of Nutrition and Biochemistry, School of Medicine, Ilam University of Medical Sciences, Ilam, Iran; 3 Nutrition and Metabolic Diseases Research Center and Clinical Sciences Research Institute, Ahvaz Jundishapur University of Medical Sciences, Ahvaz, Iran; 4 Student Research Committee, Shiraz University of Medical Sciences, Shiraz, Iran; 5 Department of Community Nutrition, School of Nutrition and Food Sciences, Shiraz University of Medical Sciences, Shiraz, Iran; 6 Health Policy Research Center, Institute of Health, Shiraz University of Medical Sciences, Shiraz, Iran; 7 Department of Community Nutrition, School of Nutrition Sciences and Food Technology), Student Research Committee (National Nutrition and Food Technology Research Institute, Shahid Beheshti University of Medical Sciences, Tehran, Iran; 8 Department of Community Nutrition, Faculty of Nutrition and Food Technology, National Nutrition and Food Technology Research Institute, Shahid Beheshti University of Medical Sciences, Tehran, Iran; 9 Infertility and Reproductive Health Research Center, Health Research Institute, Babol University of Medical Sciences, Babol, Iran

**Keywords:** Bioactive peptide, Dairy products, Bladder cancer

## Abstract

**Background:**

Bladder cancer is the sixth most common cancer worldwide. Bioactive peptides (BP) are digestion-resistant (enzymatically stable) and absorbable fragments that exert physiological functions in the body. We conducted this case-control study to examine the association between dairy-derived BPs and bladder cancer.

**Methods:**

The present case-control study (103 cases and 200 controls) was a hospital-based investigation conducted in three referral hospitals in Tehran, Iran. Dietary intake was assessed using a validated 168-item Food Frequency Questionnaire (FFQ). Intake of BPs was estimated based on dairy product consumption recorded in the FFQ. Logistic regression analysis was used to examine the association between the content of digestion-resistant BPs in dairy products and the odds of bladder cancer.

**Results:**

After adjusting confounding factors, it was observed that the odds of bladder cancer were significantly lower in the second and last tertiles (T) of total peptide intake from dairy products compared to the first tertile (T_2_: odds ratio (OR) = 0.285; 95% confidence interval (CI): 0.116–0.699, T_3_: OR = 0.130; 95% CI: 0.032–0.527). Additionally, in the adjusted model, a significant inverse association was found between other dairy-derived peptides and the odds of bladder cancer.

**Conclusions:**

The findings suggest a potential inverse relationship between milk-derived BPs and bladder cancer risk, which warrants further investigation in longitudinal or interventional studies. However, further studies are needed to elucidate the biological properties and mechanism of action of milk-derived BPs.

**Supplementary Information:**

The online version contains supplementary material available at 10.1186/s41043-025-01071-2.

## Introduction

Bladder cancer is the sixth most common malignancy worldwide and the ninth leading cause of cancer-related mortality, particularly among men [1]. Tobacco use is the most significant risk factor for bladder cancer [2]. Other established risk factors include occupational exposure to industrial chemicals, environmental carcinogens, and a family history of the disease [3]. Despite advances in cancer treatment, bladder cancer remains one of the deadliest malignancies, especially in developing countries [4].

Emerging evidence highlights the significant role of nutrition in the onset, progression, and severity of various types of cancer [5]. According to the World Cancer Research Fund (WCRF), poor nutrition may contributed to up to one-third of all cancer cases in high-income countries [6]. The impact of diet on bladder cancer is considered even more pronounced, as many metabolites are excreted through the urinary tract [7].

Dairy products such as yogurt, doogh (a traditional yogurt-based drink), and cheese are staples of the Iranian diet and are commonly consumed with most meals. These foods are rich in nutrients and bioactive constituents, which contribute to their significant health benefits [8]. However, there has been considerable speculation reading the impact of dairy consumption on cancer risk and progression.

However, studies have yet to reach a definitive conclusion on this matter. The controversy may stem from the involvement of multiple factors. For example, a meta-analysis found that moderate to high consumption of milk products was associated with a significant reduction in bladder cancer risk, whereas higher intake of whole milk correlated with an increased risk. Likely due to its higher saturated fat content [9].

Bioactive peptides (BP) are digestion-resistant (enzymatically stable) and absorbable fragments released during the digestion of various food proteins, particularly those from milk products. These peptides perform multiple physiological functions in the body [10]. Although initially inactive within native protein, once released, they are believed to offer numerous health benefits, including antimicrobial, anti-hypertensive, anti-inflammatory, and anti-diabetic effects [11–13]. Consequently, BPs are considered important components for health-promoting foods and pharmaceutical applications [14]. Despite extensive research, most studies on BPs have been conducted in vitro or in vivo. Therefore, further investigation using observational method is necessary.

In the present study, we aim to determine whether the intake of milk-derived BPs is associated with a reduced risk of bladder cancer by closely examining the dietary habits of bladder cancer patients and matched controls. To the best of our knowledge, no previous research has addressed this relationship. Therefore, we conducted this case-control study to investigate the potential association between dairy-derived BPs and bladder cancer risk.

## Methods

### Study design and sampling

This hospital-based case-control study was conducted from December 2018 to March 2020 in three referral hospitals in Tehran, Iran. The sample size was calculated based on a previous study by De Stefani et al., which reported an odds ratio (OR) of 2.35 for adherence to an unhealthy dietary pattern, with a 5% α error and 20% β error [15]. The study included 103 newly diagnosed bladder cancer patients aged over 45 years, most of whom had non-muscle-invasive bladder cancer (NMIBC), with tumors confined to mucosa or submucosa, while the remainder had muscle-invasive disease. Cases were staged according to established clinical and pathological guidelines. Dietary exposure was assessed retrospectively using a validated food frequency questionnaire (FFQ). To preserve the temporal relationship between exposure and outcome, only newly diagnosed cases were included, and their dietary habits prior to diagnosis were evaluated.). To control group consisted of 200 patients from the same hospitals, selected from those admitted for non-neoplastic conditions such as trauma, disk disorders, orthopedic conditions, and disease of the skin, ear, eye, or nose, as well as acute surgical conditions (Hospital-based controls were selected from departments unrelated to cancer or urological diseases to reduce potential overlap in disease etiology).

Exclusion criteria for cases included adherence to a specific diet, a history of other types of cancer, and prior chemotherapy before enrollment. For the control group, individuals with neoplastic diseases related to smoking or those who had undergone long-term dietary modification were excluded. Data were collected through interview with all participants. New diagnosed patients were interviewed during hospitalization, while previously diagnosed patients were interviewed during follow-up visits; the same approach was applied to controls. All cases were histologically confirmed based on tumor tissue samples.

Written informed consent was obtained from all participants. All study procedures involving human subjects were approved by the Iranian Institute of National Nutrition and Food Technology Research, and the protocol adhered to the guidelines of the Helsinki Declaration. The research ethics approval code was IR.SBMU.NNFTRI.REC.1398.010. Some details of this study have been published previously [16, 17] (Unlike previous studies that assessed general dietary patterns and nutrient intake, this study specifically focused on milk-derived bioactive peptides. To estimate individual peptide intake, we utilized an in-silico simulation model based on known milk protein sequences and established enzymatic digestion pathways. This approach allowed for more targeted exposure assessment compared to conventional nutrient-based analyses).

### Dietary and non-dietary assessments

Trained interviewers administered the questionnaires to participants during their hospital stay. Dietary intake was assessed using a validated 168-item FFQ [18]. Participants reported the frequency of consumption for each food item, and energy intake was calculated using the United States Department of Agriculture (USDA) Food Composition table. For traditional foods, the Iranian Food Composition table was employed.

Participants’ weight was measured using a digital scale (Seca, Hamburg, Germany) while wearing light clothing. Height was determined with a non-stretchable tape measure, accurate to 0.5 cm. Checklists were employed to gather lifestyle, clinical, and socio-demographic information, including age, education, marital status, family history of bladder cancer, smoking status, history of chemotherapy, and aciduricemia. Additionally, participants were asked about their use of supplements such as iron, zinc, calcium, and B complex vitamins. Physical activity over the previous seven days was assessed using a validated questionnaire [19].

### Digestion-resistant peptides

Dietary intake of BPs was estimated based on dairy food consumption recorded in the FFQ [18]. To calculate the amounts of digestion-resistant or enzymatically stable BPs from dairy products, the intake of each dairy item (in grams) was multiplied by its peptide content. These peptide contents were derived from a previous in-silico study [20]. Briefly, the enzyme action tool of the BIOPEP webserver was used to simulate the proteolysis of dairy proteins [21]. Key dairy proteins—α-lactalbumin, β-lactoglobulin, αs1-casein, αs2-casein, κ-casein, and β-casein—were digested in silico using enzymes such as chymotrypsin A, trypsin, and pepsin. The resulting peptide fragments were then analyzed using the BIOPEP database to identify active sequences. These peptides were categorized according to their biological functions (anti-diabetic, immunomodulatory, anti-hypertensive, and antioxidant), total peptide count, hydrophobicity (high or low), and peptide length (dipeptides, tripeptides, tetrapeptides, etc.) [22].

### Statistical analysis

Statistical analyses were performed using SPSS software (version 21.0; IBM Corp., Armonk, NY, USA). The Kolmogorov-Smirnov test assessed the normality of variables. Independent samples t-tests compared mean values between cases and controls, while analysis of covariance (ANCOVA) was used to adjust for energy intake. The Mann-Whitney U test compared non-parametric variables, and the chi-square test analyzed categorical variables. Logistic regression models were employed to examine the association between digestion-resistant BPs from dairy products and bladder cancer risk. The adjusted model controlled for potential confounders, including age, physical activity, BMI, energy intake, fat intake, calcium intake, family history of cancer, gender, smoking status, chemotherapy history, medication use, aciduricemia history, and supplement use.

## Results

Baseline characteristics of the study population are presented in Table 1. The mean age was significantly higher in the bladder cancer group compared to controls (65.41 ± 10.61 vs. 61.31 ± 13.15 years; *p* = 0.004). Significant differences were also observed between the groups in terms of gender (*p* < 0.001), family history of cancer (*p* = 0.003), smoking status (*p* < 0.001), chemotherapy history (*p* < 0.001), and supplement use (*p* = 0.016).

**Table 1:** The study population features between the case and control groups

Variables	Case (n = 103)	Control (n = 200)	*P*-value
Age (year)^1^	65.41 ± 10.61	61.31 ± 13.15	**0.004**
BMI (kg/m^2^)^1^	24.79 ± 4.01	25.56 ± 4.06	0.148
Physical activity (MET. hour/week)^2^	1452	1440	0.334
	(511.5–4353.0)	(270.0-3939.0)	
Gender, %^3^			**<0.001**
Male	89 (86.4)	93 (46.5)	
Female	14 (13.6)	107 (53.5)	
Education, %^3^			0.723
Illiterate	101 (98.1)	193 (96.5)	
University education	2 (1.9)	7 (3.5)	
Cancer history, %^3^			1
No	100 (97.1)	193 (96.5)	
Yes	3 (2.9)	7 (3.5)	
Family history of cancer, %^3^			**0.003**
No	95 (92.2)	198 (99.0)	
Yes	8 (7.8)	2 (1.0)	
Smoking history, %^3^			**<0.001**
No	40 (38.8)	175 (87.5)	
Yes	63 (61.2)	25 (12.5)	
Chemotherapy history, %^3^			**<0.001**
No	80 (77.7)	195 (97.5)	
Yes	23 (22.3)	5 (2.5)	
Aciduricemia history, %^3^			0.341
No	100 (97.1)	198 (99.0)	
Yes	3 (2.9)	2 (1.0)	
Medication history, %^3^			0.224
No	51 (49.5)	84 (42.0)	
Yes	52 (50.5)	116 (58.0)	
Supplement history, %^3^			**0.016**
No	95 (92.2)	164 (82.0)	
Yes	8 (7.8)	36 (18.0)	

Abbreviation: BMI, body mass index; kg, kilogram; m, meter; MET, metabolic equivalent of task

^1^ Using independent samples T-test for parametric variables and values are mean ± standard deviation

^2^ Using Mann-Whitney U test for non-parametric variables and values are median (25th -75th)

^3^ Using chi-square test for categorical variables and values are frequency (percentage)

Nutrient intake comparisons between the case and control groups are summarized in Table 2. Significant differences were observed in energy intake (*p* = 0.009), fat (*p* < 0.001), fiber (*p* = 0.042), calcium (*p* = 0.049), and consumption of dairy products including milk (*p* = 0.001), yogurt (*p* < 0.001), doogh (yogurt drink) (*p* < 0.001), cheese (*p* < 0.001), ice cream (*p* < 0.001), and creams and butters (*p* < 0.001). Additionally, the content of all digestion-resistant and BPs from dairy products was significantly higher in the control group compared to cases. These differences remained significant after adjusting for energy intake (*p* < 0.001 for all).

**Table 2:** Nutrient intake of the study population between the case and control groups

Variables	Case (*n* = 103)	Control (*n* = 200)	*P*-value	*P*-value^3^
Energy (kcal/day)^1^	2192.74 (1656.91-2970.08)	1874.40 (1458.26-2548.52)	**0.009**	-
Protein (g/day)^1^	79.69 (59.86-110.19)	72.33 (55.15–98.61)	0.100	0.081
Total fat (g/day)^1^	74.80 (52.07-100.69)	58.21 (39.67–85.03)	**<0.001**	**0.045**
Carbohydrate (g/day)^1^	297.70 (229.46-421.61)	280.68 (227.61-394.65)	0.187	**0.042**
Fiber (g/day)^1^	29.31 (20.27–41.38)	23.34 (15.10–42.80)	**0.042**	**0.032**
Calcium (mg/day)^1^	850.80 (561.54-1191.85)	976.56 (713.26-1338.87)	**0.049**	**<0.001**
Milks (g/day) ^2^	78.00 ± 18.00	148.01 ± 11.00	**0.001**	**0.001**
Yogurts (g/day) ^2^	116.00 ± 18.00	133.00 ± 11.00	**<0.001**	0.245
Yogurt drink (g/day) ^2^	64.00 ± 13.00	51.01 ± 5.00	**<0.001**	0.647
Cheeses (g/day) ^2^	29.00 ± 2.00	33.00 ± 1.00	**<0.001**	**0.012**
Ice creams (g/day) ^2^	2.00 ± 0.00	2.00 ± 0.00	**<0.001**	0.451
Cream and butter (g/day) ^2^	10.00 ± 1.00	3.00 ± 0.00	**<0.001**	**<0.001**
Total peptide (mMol/day)^2^	33.78 ± 27.65	41.83 ± 22.93	**0.007**	**<0.001**
Dipeptide (mMol/day)^2^	12.62 ± 10.31	15.71 ± 8.56	**0.006**	**<0.001**
Tripeptide (mMol/day)^2^	7.07 ± 5.81	8.65 ± 4.79	**0.012**	**<0.001**
Tetrapeptide (mMol/day)^2^	4.80 ± 3.94	5.93 ± 3.26	**0.009**	**<0.001**
Pentapeptide (mMol/day)^2^	4.34 ± 3.55	5.37 ± 2.94	**0.008**	**<0.001**
Hexapeptide (mMol/day)^2^	2.06 ± 1.69	2.57 ± 1.41	**0.006**	**<0.001**
Heptapeptide (mMol/day)^2^	0.60 ± 0.49	0.79 ± 0.42	**0.001**	**<0.001**
Peptide with more than 7 residues (mMol/day)^2^	2.25 ± 1.84	2.80 ± 1.53	**0.007**	**<0.001**
Anti-diabetic peptide (mMol/day)^2^	9.42 ± 7.70	11.72 ± 6.40	**0.006**	**<0.001**
Anti-oxidant peptide (mMol/day)^2^	1.84 ± 1.51	2.27 ± 1.25	**0.009**	**<0.001**
Anti-hypertensive peptide (mMol/day)^2^	5.46 ± 4.46	6.80 ± 3.69	**0.006**	**<0.001**
Phosphorylated serine (mMol/day)^2^	4.95 ± 4.09	6.02 ± 3.35	**0.016**	**<0.001**
Disulfide bond (mMol/day)^2^	0.56 ± 0.46	0.74 ± 0.38	**0.001**	**<0.001**
Glycosylated residues (mMol/day)^2^	0.76 ± 0.59	0.95 ± 0.49	**0.004**	**<0.001**
Immunomodulatory peptides (mMol/day)^2^	0.25 ± 0.20	0.30 ± 0.17	**0.011**	**<0.001**
Peptides with low bioactivity (mMol/day)^2^	9.92 ± 8.13	12.28 ± 6.72	**0.008**	**<0.001**
Peptides with high bioactivity (mMol/day)^2^	5.36 ± 4.39	6.64 ± 3.64	**0.008**	**<0.001**
Peptides with low hydrophobicity (mMol/day)^2^	21.30 ± 17.41	26.34 ± 14.44	**0.008**	**<0.001**
Peptides with high hydrophobicity (mMol/day)^2^	12.48 ± 10.23	15.49 ± 8.48	**0.007**	**<0.001**
Peptides with low hydrophilicity (mMol/day)^2^	17.16 ± 14.05	21.27 ± 11.66	**0.007**	**<0.001**
Peptides with high hydrophilicity (mMol/day)^2^	16.61 ± 13.60	20.56 ± 11.27	**0.008**	**<0.001**

Abbreviation: kcal, kilocalorie; g, gram; mMol, milliMoles

^1^ Using Mann-Whitney U test for non-parametric variables and values are median (25th -75th)

^2^ Using independent samples T-test for parametric variables and values are mean ± standard deviation

^3^ For adjusting the role of energy intake, ANCOVA test was used

The association between baseline variables and the odds of bladder cancer is presented in Table 3. Significant positive associations were observed for age (odds ratio [OR] = 1.027; 95% confidence interval [CI]: 1.007–1.047), fat intake (OR = 1.007; 95% CI: 1.002–1.012), family history of cancer (OR = 8.337; 95% CI: 1.737–40.019), smoking history (OR = 11.025; 95% CI: 6.193–19.627), and chemotherapy history (OR = 11.212; 95% CI: 4.119–30.525). Gender was inversely associated with bladder cancer risk (OR = 0.137; 95% CI: 0.073–0.256), as was supplement use (OR = 0.384; 95% CI: 0.171–0.860). Physical activity (OR = 1.00; 95% CI: 1.00–1.00) and energy intake (OR = 1.000; 95% CI: 1.000–1.000) showed no meaningful association. Multicollinearity among covariates was assessed using the Variance Inflation Factor (VIF), with all variables having VIF values below 5, indicating no exclusion was necessary. These results are summarized in the **Supplementary Table**.

**Table 3:** Association between baseline variables and the odds of bladder cancer

Variables	OR	95% CI	*P*-value
Age (year)	**1.027**	**1.007–1.047**	**0.007**
BMI (kg/m^2^)	0.965	0.917–1.016	0.179
Physical activity (MET.hour/week)	**1**	**1.000–1.000**	**0.003**
Energy intake (kcal/day)	**1**	**1.000–1.000**	**0.043**
Protein (g/day)	1.001	0.997–1.005	0.676
Total fat (g/day)	**1.007**	**1.002–1.012**	**0.009**
Fiber intake (g/day)	1	0.994–1.006	0.967
Calcium (mg/day)	0.999	0.999-1.000	0.206
Gender			
Male	Ref.	Ref.	**-**
Female	**0.137**	**0.073–0.256**	**<0.001**
Family history of cancer			
No	Ref.	Ref.	**-**
Yes	**8.337**	**1.737–40.019**	**0.008**
Smoking history			
No	Ref.	Ref.	**-**
Yes	**11.025**	**6.193–19.627**	**<0.001**
Chemotherapy history			
No	Ref.	Ref.	**-**
Yes	**11.212**	**4.119–30.525**	**<0.001**
Aciduricemia history			
No	Ref.	Ref.	**-**
Yes	2.97	0.488–18.063	0.237
Medications history			
No	Ref.	Ref.	**-**
Yes	0.738	0.458–1.190	0.213
Supplements history			
No	Ref.	Ref.	**-**
Yes	**0.384**	**0.171–0.860**	**0.02**

Abbreviation: OR, odds ratio; CI, confidence interval; BMI, body mass index; kg, kilogram; m, meter; MET, metabolic equivalent of task; kcal, kilocalorie; ref, reference

Significant values are shown in bold

These values are odds ratio (95% CIs)

Obtained from logistic regression

**Fig. 1:**
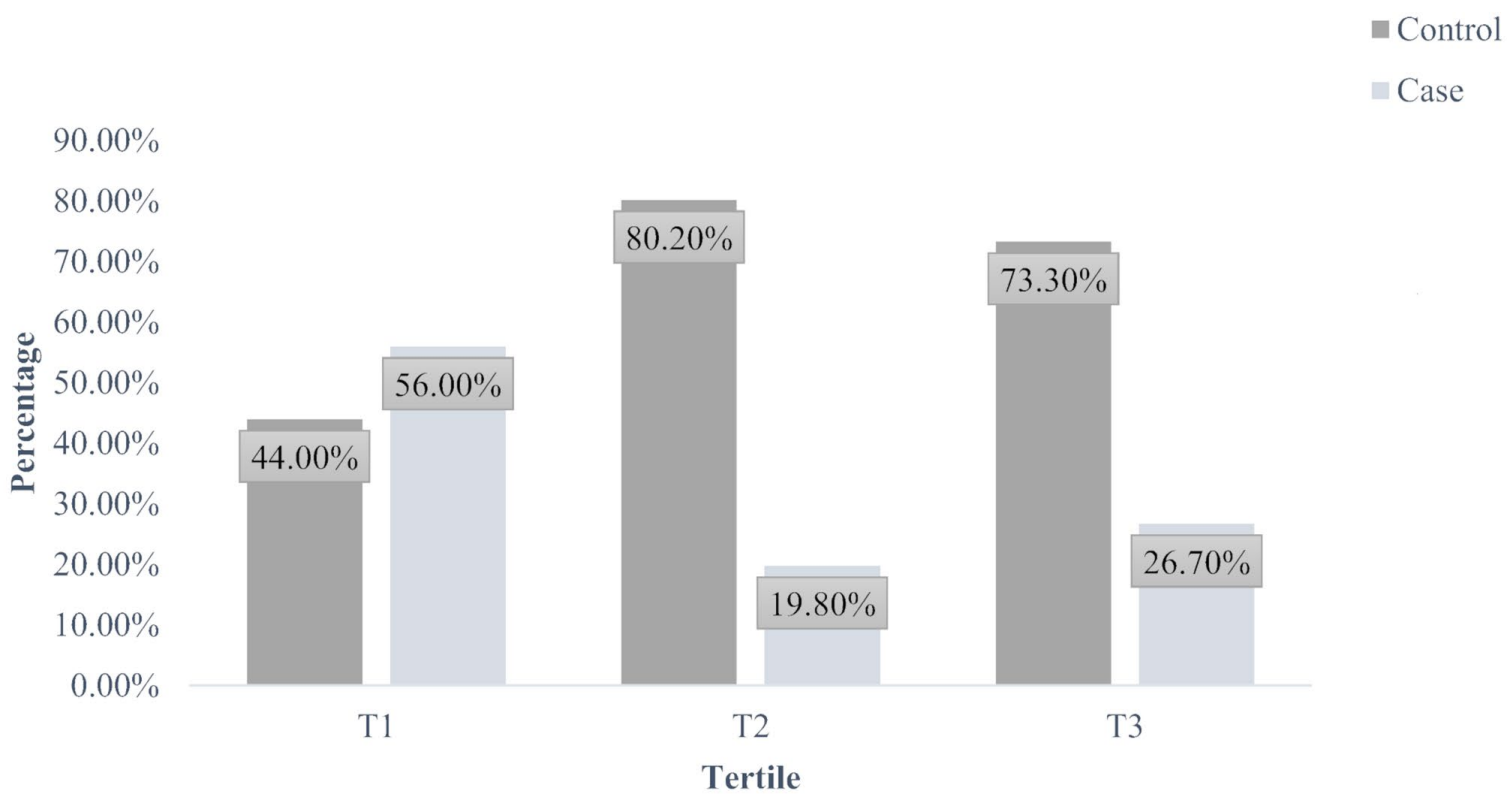
The prevalence of bladder cancer based on the tertiles of total peptides. Using the chi-square test and values are frequency (percentage). ^*^P-value was less than 0.001

The prevalence of bladder cancer according to tertiles of total peptides is illustrated in Fig. 1. The prevalence was significantly lower in both the second and third tertiles compared to the first tertile (*p* < 0.001).

The association between digestion-resistant BPs in dairy products and the odds of bladder cancer is presented in Table 4. In the crude model, individuals in the second and third tertiles showed a significant reduction in bladder cancer odds for total peptides, dipeptides, tripeptides, tetrapeptides, pentapeptides, hexapeptides, heptapeptides, peptides with more than seven residues, anti-diabetic peptides, antioxidant peptides, antihypertensive peptides, phosphorylated serine, disulfide bonds, glycosylated residues, immunomodulatory peptides, peptides with low and high bioactivity, and peptides with varying hydrophobicity and hydrophilicity (*p* < 0.001 for all). After adjusting for confounders including age, BMI, physical activity, energy intake, fat intake, calcium intake, gender, family history of cancer, smoking, chemotherapy, aciduricemia, medication use, and supplement use, all associations remained significant except for the second tertile of tripeptides, phosphorylated serine, and glycosylated residues compared to the first tertile.

**Table 4:** Association between digestion-resistant and bioactive peptides content of dairy products and the odds of bladder cancer

Variables	T_1_	T_2_	T_3_	*P*-value
		OR (95% CI)	OR (95% CI)	
Total peptide (mMol/day)				
Crude	Ref.	**0.194 (0.103–0.364)**	**0.287 (0.159–0.518)**	**<0.001**
Adjusted	Ref.	**0.285 (0.116–0.699)**	**0.130 (0.032–0.527)**	**0.002**
Dipeptide (mMol/day)				
Crude	Ref.	**0.206 (0.111–0.384)**	**0.272 (0.150–0.494)**	**<0.001**
Adjusted	Ref.	**0.290 (0.119–0.711)**	**0.097 (0.023–0.413)**	**0.001**
Tripeptide (mMol/day)				
Crude	Ref.	**0.215 (0.115-0.400)**	**0.299 (0.165–0.539)**	**<0.001**
Adjusted	Ref.	0.456 (0.195–1.096)	**0.195 (0.050–0.763)**	**0.015**
Tetrapeptide (mMol/day)				
Crude	Ref.	**0.194 (0.103–0.364)**	**0.287 (0.159–0.518)**	**<0.001**
Adjusted	Ref.	**0.285 (0.116–0.699)**	**0.130 (0.032–0.527)**	**0.002**
Pentapeptide (mMol/day)				
Crude	Ref.	**0.194 (0.103–0.364)**	**0.287 (0.159–0.518)**	**<0.001**
Adjusted	Ref.	**0.285 (0.116–0.699)**	**0.130 (0.032–0.527)**	**0.002**
Hexapeptide (mMol/day)				
Crude	Ref.	**0.228 (0.123–0.422)**	**0.284 (0.156–0.514)**	**<0.001**
Adjusted	Ref.	**0.333 (0.138–0.807)**	**0.156 (0.038–0.643)**	**0.005**
Heptapeptide (mMol/day)				
Crude	Ref.	**0.179 (0.095–0.336)**	**0.238 (0.131–0.435)**	**<0.001**
Adjusted	Ref.	**0.184 (0.072–0.471)**	**0.077 (0.019–0.317)**	**<0.001**
Peptide with more than 7 residues (mMol/day)				
Crude	Ref.	**0.215 (0.115-0.400)**	**0.299 (0.165–0.539)**	**<0.001**
Adjusted	Ref.	**0.342 (0.141–0.830)**	**0.150 (0.037–0.605)**	**0.004**
Anti-diabetic peptide (mMol/day)				
Crude	Ref.	**0.206 (0.111–0.384)**	**0.272 (0.150–0.494)**	**<0.001**
Adjusted	Ref.	**0.290 (0.119–0.711)**	**0.097 (0.023–0.413)**	**0.001**
Anti-oxidant peptide (mMol/day)				
Crude	Ref.	**0.215 (0.115-0.400)**	**0.299 (0.165–0.539)**	**<0.001**
Adjusted	Ref.	**0.342 (0.141–0.830)**	**0.150 (0.037–0.605)**	**0.004**
Anti-hypertensive peptide (mMol/day)				
Crude	Ref.	**0.186 (0.099–0.350)**	**0.262 (0.144–0.475)**	**<0.001**
Adjusted	Ref.	**0.241 (0.098–0.594)**	**0.083 (0.019–0.358)**	**<0.001**
Phosphorylated serine (mMol/day)				
Crude	Ref.	**0.215 (0.115-0.400)**	**0.299 (0.165–0.539)**	**<0.001**
Adjusted	Ref.	0.456 (0.195–1.096)	**0.195 (0.050–0.763)**	**0.015**
Disulfide bond (mMol/day)				
Crude	Ref.	**0.222 (0.121–0.410)**	**0.222 (0.121–0.410)**	**<0.001**
Adjusted	Ref.	**0.289 (0.117–0.716)**	**0.075 (0.019–0.292)**	**<0.001**
Glycosylated residues (mMol/day)				
Crude	Ref.	**0.228 (0.123–0.422)**	**0.284 (0.156–0.514)**	**<0.001**
Adjusted	Ref.	0.426 (0.176–1.028)	**0.113 (0.029–0.436)**	**0.002**
Immunomodulatory peptides (mMol/day)				
Crude	Ref.	**0.215 (0.115-0.400)**	**0.299 (0.165–0.539)**	**<0.001**
Adjusted	Ref.	**0.315 (0.129–0.768)**	**0.160 (0.038–0.674)**	**0.005**
Peptides with low bioactivity (mMol/day)				
Crude	Ref.	**0.194 (0.103–0.364)**	**0.287 (0.159–0.518)**	**<0.001**
Adjusted	Ref.	**0.285 (0.116–0.699)**	**0.130 (0.032–0.527)**	**0.002**
Peptides with high bioactivity (mMol/day)				
Crude	Ref.	**0.194 (0.103–0.364)**	**0.287 (0.159–0.518)**	**<0.001**
Adjusted	Ref.	**0.285 (0.116–0.699)**	**0.130 (0.032–0.527)**	**0.002**
Peptides with low hydrophobicity (mMol/day)				
Crude	Ref.	**0.194 (0.103–0.364)**	**0.287 (0.159–0.518)**	**<0.001**
Adjusted	Ref.	**0.285 (0.116–0.699)**	**0.130 (0.032–0.527)**	**0.002**
Peptides with high hydrophobicity (mMol/day)				
Crude	Ref.	**0.194 (0.103–0.364)**	**0.287 (0.159–0.518)**	**<0.001**
Adjusted	Ref.	**0.285 (0.116–0.699)**	**0.130 (0.032–0.527)**	**0.002**
Peptides with low hydrophilicity (mMol/day)				
Crude	Ref.	**0.194 (0.103–0.364)**	**0.287 (0.159–0.518)**	**<0.001**
Adjusted	Ref.	**0.285 (0.116–0.699)**	**0.130 (0.032–0.527)**	**0.002**
Peptides with high hydrophilicity (mMol/day)				
Crude	Ref.	**0.194 (0.103–0.364)**	**0.287 (0.159–0.518)**	**<0.001**
Adjusted	Ref.	**0.285 (0.116–0.699)**	**0.130 (0.032–0.527)**	**0.002**

Abbreviation: OR, odds ratio; CI, confidence interval; T, tertile; mMol, milliMoles; ref, reference

Significant values are shown in bold

These values are odds ratio (95% CIs)

Obtained from logistic regression

-Adjusted for variables with *p* < 0.25 in multivariate analysis according to Table 3

Adjusted model: adjusted for age (year), BMI (kg/m^2^), physical activity (MET.hour/week), energy intake (kcal/day), fat intake (g/day), calcium intake (mg/day), gender (male/female), family history of cancer (no/yes), smoking history (no/yes), chemotherapy history (no/yes), Aciduricemia history (no/yes), medication history (no/yes), and supplement history (no/yes)

## Discussion

In this study, we found that higher intake of dairy-derived BPs was significantly associated with a reduced risk of bladder cancer, an association that remained robust after adjusting for potential confounding factors.

In recent years, BPs have garnered considerable attention; however, most research has focused on isolated BPs in trial, in vitro, or in vivo settings. For example, Zho et al. engineered an anticancer fusion peptide and transfected it into ovarian cancer cells, inducing apoptosis [23]. Another study demonstrated significant anti-tumor activity in rats following administration of oligopeptide-enriched hydrolysates derived from oysters [24]. Additionally, BPs from soybeans were shown to suppress chemical carcinogens and inhibit the proliferation of skin tumor cells in mice [25].

BPs derived from dairy proteins have demonstrated significant anticancer potential in recent studies. Meisel et al. reported that casein-derived peptides exhibit anticancer activity by inhibiting cancer cell growth and stimulating immunocompetent cells [26]. Similarly, MacDonald et al. found that these peptides positively influence colon cell kinetics in vitro [27]. Roy et al. also identified an inhibitory effect of bovine milk-derived BPs on leukemia cells [28]. Lactoferrin, another key dairy component, can be processed by digestive enzymes to generate cationic peptides that promote cytotoxic activities against cancer cells via mechanisms such as cell cycle arrest, apoptosis, antiangiogenesis, antimetastasis, immune modulation, and necrosis [29]. Supporting this, a study demonstrated that lactoferrin inhibited lung metastasis and angiogenesis in mice transplanted with melanoma, lymphoma, or colon carcinoma cells [30]. The anticancer effects of BPs are often mediated through apoptosis or necrosis; in necrosis, peptides target cancer cell membrane molecules causing lysis, while in apoptosis, they disrupt the mitochondrial membrane [31]. Additionally, BPs may act as free radical scavengers, reducing lipid peroxidation and oxidative stress [32].

Recently, Parastoei et al. developed a novel method to quantify daily intake of dairy-derived BPs for the first time [10]. Building on this work, Jabbari et al. found an inverse association between dairy-derived BP intake and the risk of estrogen receptor (ER)/progesterone receptor (PR)/human epidermal growth factor receptor 2 (HER2)-negative breast cancer [33]. Inspired by these findings, our study measured the BP content of dairy products and examined its association with bladder cancer risk.

However, it is important to acknowledge that intake of milk-derived BPs is inherently linked to milk consumption. Dairy products contain other bioactive nutrients such as calcium, protein, vitamin B_2_, and fatty acids, many of which have demonstrated anticancer properties in previous studies [34–36]. Therefore, it may not be entirely accurate to attribute the observed protective effects solely to BPs, despite our efforts to adjust for these confounding nutrients in the analysis. Additionally, recent literature emphasizes other protective factors in cancer prevention and management, including the role of rehabilitation in improving patient outcomes and potentially modulating risk factors [37]. Incorporating such perspectives could offer a more comprehensive understanding of cancer prevention and management.

## Limitations

This study employs a novel measurement method to advance understanding of dietary BPs and their associations with multiple morbidities. While our approach offers greater time- and cost-efficiency compared to traditional in vitro and in vivo studies, several limitations should be noted. First, dietary data were collected using a validated FFQ, which, despite interviewer administration, may be subject to recall bias and misreporting, particularly regarding portion size and food frequency. This inherent limitation of FFQs should be considered when interpreting the findings. Also, dietary intake was assessed via FFQs, which depend on participants’ memory and honesty, making the data susceptible to recall bias and exposure misclassification. Such misclassification is likely non-differential and could attenuate the observed associations, highlighting the need for cautious interpretation of the results.

Second, given the case-control design of the present study, causal inference is limited. Although significant associations between BP intake and bladder cancer risk were observed, the temporal relationship remains unclear, raising the possibility of reverse causality. Prospective cohort studies are needed to establish temporality and confirm the direction of this association. Third, one key limitation of this study is the reliance on an in-silico simulation for estimating peptide intake, which, while innovative, remains an indirect proxy and lacks validation against real-world biological measurements. The model has not yet been compared with actual peptide levels in human samples, such as plasma or urine, following milk consumption. Future research should aim to validate the simulated intake estimations using in vitro digestion models and clinical studies that measure peptide bioavailability in biological fluids.

Fourth, the relatively small sample size and use of hospital-based controls may limit the generalizability of our results. Additionally, the observed gender imbalance between groups may have introduced sex-related bias, which should be considered when interpreting the findings. Fifth, despite adjustment for a broad set of dietary and lifestyle covariates, residual confounding remains a potential limitation of our study. Dairy products are complex food matrices containing multiple co-nutrients and bioactive compounds, including calcium, vitamin D, and probiotics, which may independently or synergistically influence bladder cancer risk. Additionally, unmeasured lifestyle factors such as physical activity intensity, sun exposure (affecting vitamin D status), or gut microbiota composition could have influenced the observed associations (Furthermore, other notable limitation of our study is the significant gender imbalance between the case and control groups. Although statistical adjustment for sex was performed, such an imbalance may lead to residual confounding or effect modification that is not fully accounted for). Sixth, subgroup analyses by bladder cancer subtype were not conducted due to limited sample size and incomplete data. Given the distinct pathophysiology and potential dietary risk profiles of subtypes, future studies should address this gap.

To our knowledge, this is the first epidemiological study to examine the association between indirectly estimated intake of dairy-derived bioactive peptides and bladder cancer risk using FFQ-based dietary data and in-silico digestion models. While innovative, this approach is inferential and requires further validation in future research.

## Conclusions

Our findings indicate an inverse association between milk-derived BPs and colorectal cancer risk; however, due to the observational nature of the study, no causal inferences can be made. Therefore, through the identification of novel compounds derived from dairy products, there is potential to discover complementary drugs for the treatment of malignancies. However, milk-derived BPs are consumed alongside other bioactive components with potential anticancer effects, such as calcium and probiotics. Despite statistical adjustments, disentangling the independent role of BPs from these co-occurring nutrients is difficult and warrants further investigation.

## Supplementary Information

Below is the link to the electronic supplementary material.

Supplementary Material 1

## Data Availability

No datasets were generated or analysed during the current study.
