# Supplementary material for: The association between digestion-resistant and bioactive peptide content of dairy products and bladder cancer: a case-control study
Source: J Health Popul Nutr. 2025 Sep 30;44:336. doi: 10.1186/s41043-025-01071-2 (PMC12486994; doi:10.1186/s41043-025-01071-2)
Supplement: Supplementary file 1 — Supplementary Material 1 [file 41043_2025_1071_MOESM1_ESM.docx]

**Supplementary Table. Collinearity statistics between variables.**

| **Variables** | **Collinearity Statistics** | |
| --- | --- | --- |
|  | **Tolerance** | **VIF** |
| Age (year) | 0.838 | 1.194 |
| BMI (kg/m^2^) | 0.871 | 1.148 |
| Physical activity (MET.hour/week) | 0.923 | 1.083 |
| Energy intake (kcal/day) | 0.274 | 3.656 |
| Protein (g/day) | 0.295 | 3.388 |
| Total fat (g/day) | 0.510 | 1.959 |
| Calcium (mg/day) | 0.701 | 1.427 |
| Gender | 0.827 | 1.210 |
| Family history of cancer | 0.721 | 1.386 |
| Smoking history | 0.770 | 1.298 |
| Chemotherapy history | 0.918 | 1.090 |
| Aciduricemia history | 0.848 | 1.179 |
| Medications history | 0.884 | 1.132 |
